# Addressing the need for improved land cover map products for policy support

**DOI:** 10.1016/j.envsci.2020.04.005

**Published:** 2020-10

**Authors:** Zoltan Szantoi, Gary N. Geller, Nandin-Erdene Tsendbazar, Linda See, Patrick Griffiths, Steffen Fritz, Peng Gong, Martin Herold, Brice Mora, André Obregón

**Affiliations:** a European Commission, Joint Research Centre, Ispra, 20127, Italy; b Stellenbosch University, Stellenbosch, 7602, South Africa; c NASA Jet Propulsion Laboratory, California Institute of Technology, Pasadena, CA 91109, USA; d Wageningen University and Research, Wageningen, 6700 AA, The Netherlands; e International Institute for Applied Systems Analysis, Laxenburg, A-2361, Austria; f ESA, Directorate of EO Programmes, Science Applications & Climate Department, Frascati, Italy; g Department of Earth System Science, Tsinghua University, Beijing, 100084, China; h Communications & Systèmes (CS), 31506, Toulouse, France; i European Centre for Medium-Range Weather Forecasts, RG2 9AX, Reading, UK

**Keywords:** Land cover, Reference data, Earth observation, Remote sensing, Applications, Ecosystem services

## Abstract

The continued increase of anthropogenic pressure on the Earth’s ecosystems is degrading the natural environment and then decreasing the services it provides to humans. The type, quantity, and quality of many of those services are directly connected to land cover, yet competing demands for land continue to drive rapid land cover change, affecting ecosystem services. Accurate and updated land cover information is thus more important than ever, however, despite its importance, the needs of many users remain only partially attended. A key underlying reason for this is that user needs vary widely, since most current products – and there are many available – are produced for a specific type of end user, for example the climate modelling community. With this in mind we focus on the need for flexible, automated processing approaches that support on-demand, customized land cover products at various scales. Although land cover processing systems are gradually evolving in this direction there is much more to do and several important challenges must be addressed, including high quality reference data for training and validation and even better access to satellite data. Here, we 1) present a generic system architecture that we suggest land cover production systems evolve towards, 2) discuss the challenges involved, and 3) propose a step forward. Flexible systems that can generate on-demand products that match users’ specific needs would fundamentally change the relationship between users and land cover products – requiring more government support to make these systems a reality.

## Highlights

• Despite the widespread importance of land cover products, current production approaches leave many end users unsatisfied.
• Frequent, global coverage of satellite imagery is now available, enabling new approaches for land cover product generation.
• New approaches and land cover products can better serve end user needs.
• A dynamic, automated land cover mapping system is proposed, with challenges outlined and their solutions proposed.
• Realization of the concept will require additional government support.

## 1 Introduction

Changes in land cover are one of the greatest and most immediate threats impacting the natural environment and then affecting the ecosystem services they provide to humans (Maxwell et al., 2016; Newbold et al., 2016, 2015; Budroni et al., 2019). These changes will continue as the population and national economies grow. To monitor and plan for these changes, and to respond to the competing land demands for bioenergy, agriculture, raw material extraction, urban infrastructure, protected areas, and other uses, accurate and updated land cover (LC) information is essential. Information on LC also guides and helps to assess progress towards various Multilateral Environmental Agreements (MEAs). For example, the United Nations (UN) has noted that LC information is fundamental to many areas of environmental monitoring and reporting, particularly in the interest of achieving the UN Sustainable Development Goals (SDGs) (Table 1). Additionally, the UN-sponsored Global Climate Observing System has highlighted the need for improved LC information (WMO, 2016), identifying a requirement for annual Earth Observation (EO)-based mapping of LC at 10–30 m resolution as part of the suite of observations called for by the UN Framework Convention on Climate Change (UNFCCC) (Table 2) and which the Paris Agreement has pledged to strengthen (UN-ESC, 2016; UN-FCCC, 2015). Moreover, LC products also play a direct role on biodiversity (Table 3) and wetlands (Table 4) protection together with combating desertification.

**Table 1:** Land cover has a direct role for seven UN Sustainable Development Goal Targets (GOFLCD, 2017; IA, 2017; UN-GA, 2015).

**Goal**	**Target**
6.6	Protect and restore water-related ecosystems, including mountains, forests, wetlands, rivers, aquifers and lakes
11.3	Enhance inclusive and sustainable urbanization and capacity for participatory, integrated and sustainable human settlement planning and management in all countries
15.1	Ensure the conservation, restoration and sustainable use of terrestrial and inland freshwater ecosystems and their services, in particular forests, wetlands, mountains and drylands, in line with obligations under international agreement
15.2	Promote the implementation of sustainable management of all types of forests, halt deforestation, restore degraded forests and substantially increase afforestation and reforestation globally
15.3	Combat desertification, restore degraded land and soil, including land affected by desertification, drought and floods, and strive to achieve a land degradation-neutral world
15.4	Ensure the conservation of mountain ecosystems, including their biodiversity, in order to enhance their capacity to provide benefits that are essential for sustainable development
15.5	Take urgent and significant action to reduce the degradation of natural habitats, halt the loss of biodiversity, and protect and prevent the extinction of threatened species

**Table 2:** Land cover has a direct role for the UN Framework Convention on Climate Change (UN-FCCC, 2015).

**Article**	**Action and Relevance**
4	Requires preparation of nationally determined contributions (requires good LC information)
5	Encourages mitigation for emission reduction 5.1. “Parties should take action to conserve and enhance, as appropriate, sinks and reservoirs of greenhouse gases as referred to in Article 4, paragraph 1(d), of the Convention, including forests.” 5.2. Specifically mentions “reducing emissions from deforestation and forest degradation, and the role of conservation, sustainable management of forests and enhancement of forest carbon stocks, as well as “the importance of incentivizing, as appropriate, non-carbon benefits”
7	Discusses adaptation, 7(c) specifically requests strengthening of systematic observations, which include 10-30m annual LC maps.
10	Requires aspects of technology development and transfer as LC is a key factor in determining stocks and emissions
13.7	Requires emissions reporting. “Each Party shall regularly provide the following information: (a) A national inventory report of anthropogenic emissions by sources and removals by sinks of greenhouse gases… (b) Information necessary to track progress made in implementing and achieving its nationally determined contribution under Article 4.”
13.8	Encourages climate change impact reporting

**Table 3:** Land cover has a direct role for the Convention on Biological Diversity (CBD, 2016, 2010).

**Target**		**Generic Indicator**
5	The rate of loss of all natural habitats, including forests, is at least halved and where feasible brought close to zero, and degradation and fragmentation is significantly reduced.	Trends in: extent of forest and other natural habitats; fragmentation of forest and other natural habitats; degradation of forest and other natural habitats; extinction risk and populations of habitat specialist species in each major habitat type
7	Areas under agriculture, aquaculture and forestry are managed sustainably, ensuring conservation of biodiversity.	Trends in: proportion of area of agriculture under sustainable practices; extinction risk and populations of agro-ecosystem associated species; proportion of area of forest production under sustainable practices; extinction risk and populations of forest-specialist species in production forest
11	At least 17% of terrestrial and inland water areas, and 10% of coastal and marine areas, especially areas of particular importance for biodiversity and ecosystem services, are conserved through effectively and equitably managed, ecologically representative and well connected systems of protected areas and other effective area-based conservation measures, and integrated into the wider landscapes and seascapes.	Trends in: areas of particular importance for biodiversity conserved; areas of particular importance for ecosystem services conserved; connectivity and integration of conserved areas
14	Ecosystems that provide essential services, including services related to water, and contribute to health, livelihoods and well-being, are restored and safeguarded, taking into account the needs of women, indigenous and local communities, and the poor and vulnerable.	Trends in: safeguarded ecosystems that provide essential services; extinction risk and populations of species that provide essential services; benefits from ecosystem services; restoration of ecosystems that provide essential services
15	Ecosystem resilience and the contribution of biodiversity to carbon stocks has been enhanced, through conservation and restoration, including restoration of at least 15% of degraded ecosystems, thereby contributing to climate change mitigation and adaptation and to combating desertification	Trends in carbon stocks within ecosystems

**Table 4:** Land cover has a direct role for the UN Convention to Combat Desertification and the Ramsar Convention on Wetlands (Ramsar Convention, 2015; UN-CCD, 2013, 2007).

**UN Convention to Combat Desertification**		
**Strategic Objectives**		**(Progress) Indicator**
At CoP 12, country Parties agreed to link the implementation of the Convention to the SDGs in general, and Target 15.3 on Land Degradation Neutrality in particular		15.3.1--Percentage of land that is degraded over total land area
To improve the condition of affected ecosystems		Trends in land cover; trends in land productivity or functioning of the land
To generate global benefits through effective implementation of the UNCCD		Trends in carbon stocks above and below ground

**Ramsar Convention on Wetlands (Selected Targets)**		
6	There is a significant increase in area, numbers and ecological connectivity in the Ramsar Site network, in particular under-represented types of wetlands including in under-represented ecoregions and Transboundary Sites.	
8	National wetland inventories have been initiated, completed or updated and disseminated and used for promoting the conservation and effective management of all wetlands.	
12	Restoration is in progress in degraded wetlands, with priority to wetlands that are relevant for biodiversity conservation, disaster risk reduction, livelihoods and/or climate change mitigation and adaptation.	

Although more and better LC information is available than ever before, the available products simply do not meet many users and applications’ needs (Herold et al., 2011; Bontemps et al., 2012; Tsendbazar et al., 2015, 2017). This results in a variety of important impacts, including unmet MEA reporting requirements, compromised ability to monitor and manage change in a timely manner, protect biodiversity, or combat desertification, and a reduction in the accuracy with which we can predict climate change and its impacts (Bontemps et al., 2012; Joppa et al., 2016; O’Connor et al., 2015).

The core of this problem is that the range of user needs and applications varies widely, yet there is currently no practical way to generate products that can meet those varied needs. For example, different users have different requirements for the number and types of LC classes, spatial resolution, temporal resolution, and geographic scope. This wide variance in user needs is demonstrated in Table 3 where, for example, the requirements for biodiversity indicators are very different from those of other fields such as water resource management or agricultural monitoring. Such varying user needs for environmental data are not restricted to LC (e.g., van Dijk, 2014; Moomaw et al., 2017). Although some excellent routinely produced LC products exist (e.g., from the MODIS sensor and the Copernicus Land Monitoring Service), such standard products lack flexibility. Many LC products are also produced on an ad hoc basis with specific user needs in mind, but while satisfying the needs of the target users the products may be only of limited use to a wider audience. The need for consistency - from country to country, or over time so that LC change can be tracked - is a related problem. For example, the UN Statistical Commission’s group on SDG indicators needs to report on LC-related indicators with global coverage using consistent methods on an annual basis, and the OECD requires globally consistent information to support the development of appropriate policy guidance. However, national governments need consistency only within their national boundaries and their needs, such as the classes used or their definitions, often differ from those of the UN or OECD. Clearly, a “one size fits all” approach is inadequate.

Meeting the needs of the many and varied users that require LC information points to moving from the current situation towards a flexible approach for generating LC products on-demand and according to user and policy needs. The massive amounts of EO data that are now openly available and the rapidly decreasing cost of processing are key factors that can enable this move. Here we review the current situation in EO data repositories (both satellite imagery and reference data) and processing environments and then discuss ways to establish an integrated and flexible LC monitoring system and the challenges in doing so.

## 2 The current state of EO data repositories, platforms and research infrastructures

### 2.1 Earth observation data repositories

Access to very large numbers of images is critical to developing accurate, useful, and timely LC datasets. The amount of freely available (i.e., no-cost) EO data available today is unprecedented due to the open data policies that have been adopted within the last decade by United States Geological Survey (USGS) and NASA for Landsat, MODIS, and other sensors, and by the European Space Agency (ESA) and the European Commission (EC) for the Copernicus programs. Even so, important gaps in coverage remain, particularly in cloudy regions such as the biodiversity-rich – and rapidly changing – tropical rainforests. Furthermore, accurate detection of LC change – one of the key applications of LC data – requires extensive image time series to provide sufficient accuracy; so the need for more data continues.

Fortunately, other space agencies are opening up their archives as well. China, for example has made data from several land observation satellites publicly available [http://www.cresda.com/EN/], and the Canadian Space Agency and the Canada Centre for Mapping and Earth Observation are making about 36,500 RADARSAT-1 synthetic aperture radar images available to the public at no cost. Initiatives like these will help fill gaps in the global image archives, especially in areas where cloud cover is a limiting factor for optical imagery. While all the above-mentioned missions deliver appropriate data capable of supporting LC mapping as well as monitoring, it should not be assumed that key satellite missions and supporting archives will always have the government support needed to maintain continuity in acquisition and access; continued support for these activities is crucial (Buchanan et al., 2018).

The Copernicus program is currently the largest producer of freely available EO data globally. The Sentinel-1 radar mission, the Sentinel-2 high spatial resolution optical mission and the Sentinel-3 optical mission are now operational, each with a twin platform constellation. The Sentinels currently produce about 25 TByte of observation data each day, disseminated through ESA’s primary distribution channels (i.e., the Sentinel Data Hub) [https://scihub.copernicus.eu/dhus/]. For Sentinel-2AB, which likely has the highest relevance for LC mapping applications, routine production of global surface reflectance (L2A) data began in early 2019 (Szantoi and Strobl, 2019). The global Landsat archive holds more than 6 M unique scenes that provide more than 40 years of data, and higher-level science products such as surface reflectance are available through on-demand interfaces (Wulder et al., 2019). In recognition of the value of Analysis Ready Data (ARD), which is gridded to a standard tile size, atmospherically corrected and complemented with masks for clouds, cloud shadows, snow/ice, etc., ARD is now produced routinely for the US land area and global ARD production is being planned (Siqueira et al., 2019).

Private EO imaging activities have also greatly increased in scope and imaging capacity. For example, Planet Labs’ Dove microsat constellation acquires global images daily, and ICEYE is setting up a constellation of small SAR satellites. However, although these missions will be valuable, especially in combination with calibrated science missions such as the Sentinels, their data comes under a commercial licence model and thus their impact on future LC mapping may be limited.

In addition to satellite imagery, reference data are needed for training LC classification algorithms and validating LC maps. Reference data are often collected by visually labelling LC types using very high resolution satellite images. Reference data can also be collected in situ but the cost is considerably higher than visual interpretation. In fact, access to high quality reference data is often the limiting factor to generating useful LC datasets. The coordinated international program Global Observation for Forest and Land Cover Dynamics (GOFC-GOLD) promotes the sharing of reference data through their portal, though this represents only a small fraction of the data available. Reference data have been collected through Geo-Wiki, a tool for crowdsourcing land cover data from very high resolution satellite imagery (Fritz et al., 2017; Laso Bayas et al., 2017), and published in Pangaea, an initiative holding over 16 million geospatial measurements (https://www.pangaea.de) including other open reference datasets. The online tool called LACO-Wiki (See et al., 2017) links reference datasets to LC, land use and change monitoring (LandSense Citizen Observatory), while novel approaches are being developed for collecting multi-purpose reference data as part of the Copernicus Global Land Monitoring Service (CGLS) (Tsendbazar et al., 2018). All these data, once assessed and validated, will become freely available. GEO, perhaps through its emerging Knowledge Hub concept, may be an appropriate organization to facilitate linking shared reference databases together. As regional level examples, the European Soil Bureau Network (Montanarella et al., 2005) and Eurostat’s Land Use/Cover Area frame Survey (LUCAS, https://ec.europa.eu/eurostat/web/lucas/data/database, Gallego and Delincé, 2010) provide high-quality reference datasets. They are sampled at a regular predefined 2 km grid, have been surveyed at three-year intervals since 2006 and provide a wealth of information on LC, land use and topsoil properties. They have been proved to be extremely valuable in EO-based LC mapping exercises after some metadata-related filtering. As such, they have been employed for continental LC mapping in Europe (Pflugmacher et al., 2019) and for validating LC products (Karydas et al., 2015). More recently, the BigEarthNet initiative emerged, which contains almost 600 K image patches from Sentinel-2 labelled using CORINE LC 2018 for 10 European countries (http://bigearth.net/). While all of these activities are helpful, access to adequate reference data will remain very challenging in many parts of the world, particularly in developing nations or remote or chronically cloudy areas. The situation is further complicated because reference data gradually goes out of date and must be updated.

Ground-based data infrastructures such as the United States’ Long Term Ecological Research (LTER) sites and the National Ecological Observatory Network (NEON) and Australia’s Terrestrial Ecosystem Research Network (TERN) are often not considered by LC producers as a source of reference data yet they represent an underexploited opportunity for integrating data from multiple sources such as these.

### 2.2 Earth observation processing environments

Today the main EO data repositories (i.e., Sentinel and Landsat) are mirrored in commercial cloud environments in addition to the archives at their parent organizations. For example, Amazon Web Services (AWS) stores copies of both the global Sentinel and Landsat archives, and makes them available for cloud-based processing. Similarly, Google Earth Engine links to mirrored EO data in the Google cloud and provides highly scalable processing capabilities. In Europe, the EC has initiated five DIAS (Data and Information Access Services) systems. These distributed storage and processing environments are designed to simplify access to Copernicus data. Currently most DIAS systems host and provide access to the main EO data repositories, which are co-located within a distributed processing environment. Most also provide access to a range of EO tools and offer a variety of pricing models (e.g., pay per use, subscription). The commercial EO data repositories hold data from various sources, which allows the users to generate complex and/or continuous (time) datasets after harmonization, which is often done by the repository provider.

ESA has initiated several Thematic Exploitation Platforms (TEPs, https://tep.eo.esa.int/), each dedicated to an EO thematic area such as Forests or Food Security, offering a wide range of predefined tools specifically targeted at non-EO expert users. The private EO industry is increasingly offering cloud-based EO services, for example, the Sentinel-hub (https://www.sentinel-hub.com) is now receiving 2 million requests to their cloud-based service per week. It provides predefined tools for visualization and the possibility to script user-defined processing steps. Planet (www.planet.com), which operates a large constellation of Earth-imaging satellites, also offers an automated, cloud-based imagery and analytics platform.

As the number of platforms providing access to EO data increases, so does the importance of data product standards and, more broadly, interoperability. This is because many products will require data sourced from different providers and, without standards, users will be forced to work with a cumbersome number of independently developed systems. Fortunately, the largest satellite data providers are already moving in this direction (Siqueira et al., 2019). For example, NASA now provides a Harmonized Landsat and Sentinel-2 (HLS) surface reflectance dataset for North America as well as some globally distributed areas at 30 m, while ESA is working on a pre-operational demonstration service, where they harmonize and fuse Sentinel-2 (S2) and Landsat 8 data at a common 10 m resolution. Such data can be stored in various locations such as data cube infrastructures (Strobl et al., 2017) while connected and processed through cloud-based systems such as AWS and DIAS.

Cloud-based platforms can host a variety of services such as the generation of derived products, or tools for data visualization, exploration and application. Although these can be developed by commercial entities, the costs involved often prohibit access to many users, particularly but not exclusively for developing countries. Development and hosting by government or international organizations can greatly increase access to utilize the data. For example, the UN Food and Agriculture Organization’s (UN-FAO’s) SEPAL (System for Earth observations, data access, Processing and Analysis for Land monitoring) is an open cloud-based system focused on monitoring and reporting on forest areas that is geared towards developing countries. Developing and sustainably hosting such services by national and international organizations is crucial to guarantee reliable access to data and tools that are needed to utilize and apply the data to address societal problems. Government/commercial hybrids (e.g., DIAS) can also work but government support, especially in developing countries, will usually be needed.

## 3 Towards customized, on-demand land cover information products

The wide range of LC products needed by users can best be met by processing systems able to accommodate user-defined tailoring via user-provided parameters – for the number, types and definition of classes, area of interest, and temporal needs – and then generate, on-demand, the desired, customized product. While this has some challenges it is technically feasible, and here we explore the key characteristics of such systems as well as the challenges in developing and operating them.

Such on-demand products would provide a valuable means for consistent national reporting across regions. Achieving this requires open data policies and freely accessible EO data archives that provide the necessary observation data at suitable temporal frequency, and spatial and radiometric resolutions to perform automated mapping. Because of rapidly increasing EO data volumes and the need to process huge, multi-temporal datasets, the conventional model of downloading EO data to desktop environments is no longer feasible.

### 3.1 Customized land cover products: user-driven systems

One of the biggest hurdles to generating user-needs-specific LC products is that it is, traditionally, a labor-intensive process. Generating customized on-demand LC products, as well as consistent, periodic ones, requires automation, and although automated LC product generation itself is not new – NASA has been generating regularly updated LC products using MODIS data for years – generating customized products requires a somewhat different approach. Advances in science and technology now make such systems possible and some of these advances are starting to be incorporated into existing systems. For example, USGS has developed a system called LCMAP that will largely replace the current approach for generating national (USA) LC products, using freely available Landsat (4, 5, 7 and 8) data. The system is capable of using the entire Landsat archive (∼40 years) to generate land cover and land surface change products, and further support our call for the use of Analysis Ready Data (Brown et al., 2020) since Landsat ARD is the fundamental data type for their processing chain. Tsinghua University in China is also developing a LC product generation portal that will allow users to specify the characteristics of the product they need by defining their own classification legend and samples through an interface and perform image classification with data available on the portal, or use a combination of user-collected samples and pre-collected ones stored on the portal to produce new LC products. The Copernicus Global Land Service (CGLS) in Collection 2 adopted the production of fractional cover layers to complement the core global land cover products. These fractional cover layers can now be utilized and combined in order to create tailored and customized categorical land cover maps based on users’ individual legend requirements (Buchhorn et al., 2019). These activities are an excellent start, but more is needed.

For example, while international organizations might be able to utilize such services, national governments are more likely to trust, adopt and report data that they produce themselves (Saah et al., 2020), even if the processing environment may be supplied by others. Such is the case for the Colombian Data Cube (Ariza-Porras et al., 2017), which is supported by the System Engineering Office of the Committee on Earth Observation Satellites (CEOS), but gives the flexibility (data ownership, extensibility, lineage, replicability) to develop the tailored algorithms some users require. The Data Cube concept provides a context for the user-driven capabilities discussed in this paper, and as Data Cube installation becomes easier (Giuliani et al., 2020), it has the potential to facilitate further implementation of these capabilities.

**Fig. 1:**
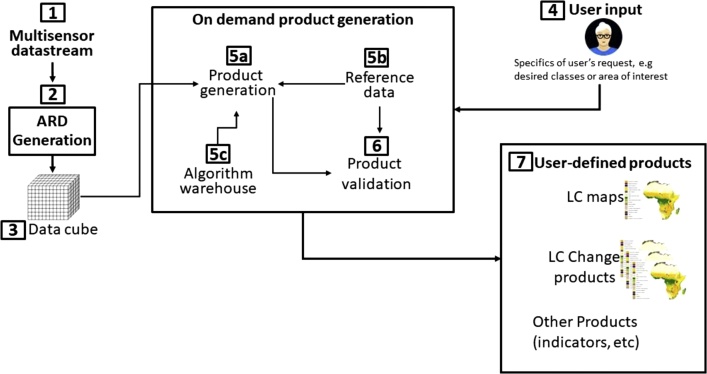
Major components of the generic architecture and the basic flow of data between them. (1,4) Inputs: Data are freely available from several sensors, user provides request details such as types and number of classes, the area of interest, temporal information, and spatial resolution. (2,3) Preprocessing: Data are processed into a consistent, normalized, pixel-based format (ARD – CARD4L) so all pixels are comparable; these can be stored in a “data cube” environment to facilitate efficient access to time series and eliminate the data processing burden. The (5a) product generation then requires (5b) reference data for training and validation and is supplied either by the user or a shared reference database. (5c) A variety of algorithms can be made available from an open source “algorithm warehouse”. (6) Validation: The product is assessed for accuracy by comparing with independent validation reference data. (7) Products: the approach can support a wide variety of other products by using different algorithms.

Fig. 1 shows a generic architecture that an automated system capable of generating customized products might utilize. The challenges in developing and operating such a system are discussed in the next section.

### 3.2 Challenges to dynamic land cover generation

Developing and operating a system such as that in Fig. 1 faces a number of challenges. These are discussed here and summarized in Table 5.

**Table 5:** Challenges and suggested solutions in setting up an on-demand LC monitoring system.

**System comp.**	**Challenge**	**Solution**	**Alternatives**
Imagery data	Uninterrupted flow and quality	Free and harmonized datasets/sensor data streams (e.g., Copernicus, USGS)	Private providers (but with cost implications)
	Interoperability between data cubes of different infrastructures	CEOS established requirements on ARD generation	
	Global coverage without gaps		

Reference data	High quality and up to date	Use citizen science and crowdsourcing for data collection	Funded program for data collection
	Open access	Advocate for continuously updated, peer reviewed reference datasets	Better exploitation of data from ground-based observation networks
	Curation & interoperable repositories	Harmonize nomenclatures among collections	Exploit new machine learning for generation of synthetic training data
	Initiatives and incentives to share data	Create incentives for data sharing	Strengthen LC mapping approaches that are independent of training data
	LC definitions		
	Standardized data formats, metadata and collection protocols		Develop business models exploiting commercial value of reference data

Analysis Ready Data	Processing and storage cost	Harmonized cloud storage	Local storage with access rights
	Easy access to different clouds	On-demand creation of ARD	

User input (requirements)	Based on policy needs	Engagement of national and international policy institutions	Pre-set policy targets

Processing	Must be robust and encompass a wide range of algorithms	Internationally accepted (published), open source, policy-targeted	Developed by institutions, tuned for policy targets
	On-demand production in cloud environment	Interoperability and federation of processing and analytic environments	Paid access

*Reference data*. The biggest challenge is the availability of high-quality reference data, which are required to train the machine learning algorithms (so it can properly identify the various classes of interest) and to assess product accuracy. There are four aspects to address:

1 Many areas simply lack enough data for the generation of high-quality products. These gaps will limit the geographic (and temporal) coverage that an on-demand system can provide; unfortunately, many of the existing gaps prevail in areas of high biodiversity and rapid change.
2 Accessibility of reference data is a key issue, sometimes relating to privacy-related constraints (e.g., with the land parcel reference data for agricultural subsidies in the EU) but often simply the unwillingness to share labor-intensive datasets.
3 LC definitions are not harmonized among the different reference sources, e.g., the definition of “forest” varies among countries and organizations. This happens because reference data are usually collected for specific applications and developers use their own nomenclatures. Part of this problem is also the lack of harmonized protocols for new reference data collections.
4 The lack of systematic data curation mechanisms is another key issue. Large quantities of reference datasets exist on old storage media scattered across research laboratories or even on paper from field surveys. A centralized, long-term data curation environment for reference and training data with standardized data formats, access and metadata is a critical requirement in this context.

An important step forward that will address these challenges is to start developing an open, shared, global database of reference data (WMO, 2016). In many cases, existing reference data were collected with government support but without a corresponding requirement by funding agencies that the data be made publicly available – a policy issue that funding agencies need to address and that would further development of an open reference database. Additionally, the Group on Earth Observations (GEO) has developed a set of Data Sharing Principles that all of its 200+ member countries and participating organizations have endorsed, providing another entry point to facilitate the development of open systems. While an open reference database will not emerge overnight, its incremental growth will gradually expand the geographic coverage available for automated LC products; governments and international organizations need to support its development.

#### 3.2.1 Data volume

Data volume will be a challenge when LC products are needed for large or global areas, as this can result in petabyte-scale mapping exercises. These not only stress the computing resources needed for processing and storage, but they also stress the infrastructure used to move those data to where they are needed. Interoperability and federation between computing environments are key challenges in this context. Both of these can be a critical obstacle for developing countries wishing to generate their own products.

#### 3.2.2 Availability of satellite images

Although more images are now being acquired than ever before, there are gaps in the openly accessible observation record, particularly for the humid tropics where cloud cover often obscures the view of optical instruments like Landsat and Sentinel-2, currently the source of most of the optical image data. These gaps will limit the geographic scope that a system like that in Fig. 1 can support. However, radar data, for example from the German Terra-SAR-X mission, would be valuable if opened up more broadly to the EO community because it is unaffected by clouds. There are also many historical gaps, particularly in the early years of satellite Earth Observation. Gaps do not mean that data were never acquired – in fact, more than 30 sovereign states have financed satellites with global LC observing capabilities, and around 200 have been successfully launched since 1972 (Belward and Skøien, 2015). However, the archives from many of these missions are not openly accessible and thus not available to LC processing systems (Wulder and Coops, 2014). GEO has a role to play here, encouraging, as it has done for some time, its member countries to open their satellite image archives and improve access.

#### 3.2.3 Standardized data formats

Data providers tend to use their own formats, complicating processing by adding a pre-processing step. A good start in this direction is agreement among some of the large space agencies within CEOS who will attempt to harmonize some of their imagery data based on commonly agreed specifications; this agreement has led to the so-called CEOS Analysis Ready Data for Land (CARD4L) products (Lewis et al., 2018).

#### 3.2.4 Curation and standardization of reference data

While data repositories for EO data or high-level products exist, reference and training data repositories are only beginning to emerge. Urgently needed are centralized data curation environments where reference data is made accessible and stored on a long-term basis. Important prerequisites are standardized dataset specifications, formats and metadata that would allow interoperability and discoverability across such environments.

#### 3.2.5 Utilizing advancing technology

Finally, new methodological approaches from machine learning and other areas of artificial intelligence need to be explored and tailored to the requirements for dynamic LC mapping. For example, deep learning and transfer learning can unravel novel mapping capabilities and can greatly improve the capability of mapping across temporal and spatial scales. New machine learning approaches (e.g., Generative Adversarial Networks) have also shown exceptional generalization capacities potentially allowing the creation of synthetic training and reference data. These methodological approaches can also provide valuable mechanisms for fusing different types of data such as optical and SAR data. For example, data from the Sentinel-1 SAR Mission could be fused with the Sentinel-2 optical data record over cloud-prone areas (He and Yokoya, 2018).

### 3.3 Additional products and services through a land cover mapping system

Beyond LC, governments need a broad range of other products and services such as progress indicators relevant to the SDGs and other MEAs, decision support products to assist countries in meeting their UNFCCC, CBD or UNCCD obligations, and information to bolster the bio-economy or help guarantee food security. In response, flexible systems that can generate a wide variety of products and services are beginning to be developed; DIAS, mentioned earlier, is a good example. This is important because systems like DIAS represent a cost-effective way to provide operational homes for LC processing. Another important activity is the CEOS Open Data Cube mentioned earlier. The Open Data Cube is a scalable system with an architecture somewhat similar to that shown in Fig. 1 but with the specific goal of minimizing the technical skills needed to install and operate it. Under development now, the intent is to democratize access to both the technology and the product generation algorithms, thus simplifying access to important production capabilities for countries and other users that may otherwise lack sufficient capacity. In addition to LC, the Open Data Cube is incorporating algorithms related to water detection, water quality, landslide risk, greenness, and other areas. ESA is engaging in several efforts to stimulate new cloud-based EO services that provide a high degree of interoperability (‘federation of platforms’) and to provide the user community with a stimulating environment that fosters co-creation and collaboration in addition to offering scalable and efficient processing solutions.

### 3.4 Development paths to achieving customized, on-demand land cover information products

Conceptually there are two types of pathways towards the user-driven systems discussed here. A top-down approach would consist of a large, coordinated network, perhaps analogous to that used for weather forecasting and managed by the World Meteorological Organization. Initiating and implementing this approach would be quite challenging due to its scope, cost, and the level of international coordination effort required. Currently, the authors are unaware of any discussions focused on developing such a system.

An approach with a large bottom-up component is more practical. For example, individual entities would develop their own system with the geographic scope and computational support appropriate to meet their own needs (see the previously mentioned efforts: LCMAP/USA, Copernicus Global Land/EU or global, LC product generation portal/China). These entities could be national governments, but also could be NGOs, sub-national governments, or collaborative efforts of any size or mix of organization types. The Data Cube approach could prove very useful here.

On a more technical level, important developments include those on interoperability and federation of computing environments, standardization of data and metadata as well as initial efforts working towards reference data standardization and curation platforms. Examples for these crucial elements include the web interface standards from the Open Geospatial Consortium (OGC), the ESA Exploitation platform Common Architecture (https://eoepca.github.io/), the STAC metadata specification (https://stacspec.org/) and the radiant.earth training data repository (https://www.mlhub.earth/).

In the long run it is possible that such an organically grown approach could expand to something like the large network mentioned earlier. Hybrid approaches that combine bottom-up expansion with some top-down coordination are also possible.

## 4 Conclusions

Although many MEAs and societal applications depend on up-to-date, high quality LC information, systems that can generate on-demand LC products that are customized for this wide range of users are not yet operationally available. This constrains society’s ability to monitor and respond in a more targeted and focused manner to the important changes occurring in land areas across the globe. Advances in both science and technology now make the development of automated systems possible that can address this shortcoming. Solutions for critical technical challenges towards the goal of truly dynamic LC mapping are now gradually emerging or maturing. While some challenges remain for developing countries or cloudy areas in terms of EO data availability, access and processing these can be gradually addressed and thus allow LC product generation anywhere on Earth. Developing such user-driven systems will fundamentally change the relationship between users and LC data, but government support will be needed to make them a reality. Given the central importance of LC information to society, this should be a priority.

## Funding

The research described in this paper was in part carried out at the Jet Propulsion Laboratory, California Institute of Technology, under contract with the NASA; government sponsorship is acknowledged.

## CRediT authorship contribution statement

**Zoltan Szantoi:** Conceptualization, Writing - original draft, Writing - review & editing. **Gary N. Geller:** Conceptualization, Writing - original draft, Writing - review & editing. **Nandin-Erdene Tsendbazar:** Writing - original draft, Writing - review & editing. **Linda See:** Writing - original draft, Writing - review & editing. **Patrick Griffiths:** Writing - original draft, Writing - review & editing. **Steffen Fritz:** Writing - original draft. **Peng Gong:** Writing - original draft. **Martin Herold:** Writing - original draft. **Brice Mora:** Writing - original draft. **André Obregón:** Writing - original draft.

## Declaration of Competing Interest

The authors declare that they have no known competing financial interests or personal relationships that could have appeared to influence the work reported in this paper.
